# An Integrative Neuropsychological Approach to Chronic Pain, Emotions, and Clinical Symptoms

**DOI:** 10.1155/2023/9786372

**Published:** 2023-12-31

**Authors:** Carmen M. Galvez-Sánchez, Lorys Castelli, Casandra I. Montoro

**Affiliations:** ^1^Department of Psychology, University of Jaén, Jaén, Spain; ^2^Department of Psychology, University of Turin, Turin, Italy

Chronic pain is a serious and increasing health problem, frequently accompanied by high comorbidity of anxiety, depression, insomnia, and cognitive disorders [1–4]. It is estimated that the prevalence of chronic pain in Europe is 19% [5, 6]. In 2006, Breivik et al. [4], prevented a prominent increase in chronic pain proportional to the population aging. Indeed, this prominent increase would entail higher socio-health costs and would require more effective strategies for its prevention, diagnosis, and treatment [4]. Nevertheless, chronic pain itself negatively affects the quality of life of patients and patients' relatives and generates high socio-sanitary expenses. The pain-related high socio-sanitary expenses are the result of the efforts on pain management and numerous indirect economic costs (e.g., by the labour productivity lessen and absenteeism and disability heighten) [4–6] Given the above-mentioned aspects, chronic pain has been recognized as a bioethical issue and different international organizations (e.g., World Health Organization and International Association for the Study of Pain) have declared the exigency of an adequate pain therapy as a human right [6]. To meet adequate pain therapy requirements, an integrated approach to neuropsychological, emotional, and clinical symptoms is essential. In this detail, the transdiagnostic and personalized perspectives have been scientifically and clinically supported [7, 8].

The special issue “An Integrative Neuropsychological Approach to Chronic Pain, Emotions and Clinical Symptoms” aimed at highlighting the latest progress on chronic pain's neuroscience and related symptoms to promote prevention and intervention-integrated strategies. This research topic includes five original research articles. The majority (three in total; [9–11]) examined fibromyalgia syndrome (FMS), one of the prototypical chronic pain conditions characterized by widespread chronic pain and symptoms, such as fatigue, unrefreshing sleep, cognitive deficits, and comorbid emotional alterations (i.e., anxiety and depression) [12, 13]. Concerning cognitive deficits, FMS patients tend to exhibit difficulties in verbal memory, organization and planning abilities, strategic planning, self-regulation, processing speed, attention, and cognitive flexibility [9–11]. The remaining studies explored chronic tension-type headache (CTTH) and rats with global cerebral ischemia [14, 15]. The last one, investigated the effect of voluntary wheel running on striatal dopamine levels and anxiety-like behaviour in rats with global cerebral ischemia. This study is not included in the present editorial as the results and methodology are difficult to be generalized and replicated in humans [15].

The first study [9], investigated executive functions in FMS patients compared with healthy controls via a Go/No-Go task. The variability of reaction time (RT)—by using traditional and ex-Gaussian parameters—was evaluated as a marker of executive function impairments. The study demonstrated that indices of RT variability, in particular those derived from the ex-Gaussian function, maybe a complement of speed and accuracy parameters in the assessment of executive function impairments in FMS and, therefore, facilitate the personalization of cognitive function therapies. In particular, compared with controls, FMS patients showed greater intraindividual RT variability along the task (indexed by the ex-Gaussian parameter tau) and a heightened decrease in the Go/No-Go hit rate after the change of the task rule (i.e., No-Go stimuli response). No group differences were observed regarding the false alarm rate. Results suggested deficient cognitive flexibility (decline in hit rate after the task change) but intact inhibitory response (no false alarm differences) in FMS. Moreover, increased tau in FMS indicated greater fluctuations in executive control and more frequent temporary lapses of attention.

The second study [11] assessed the negative impact of FMS-associated symptoms on the functional capacity of the patients. The FMS functional capacity was measured by the Fibromyalgia Impact Questionnaire (FIQ). The FIQ scores were positively associated with the majority of patients “symptoms (i.e., pain, fatigue, insomnia, depression, and trait anxiety). Among these symptoms, depression, fatigue, and pain catastrophizing (in this order) were those with more predictable power of FMS functional capacity. Furthermore, the most relevant factors affecting the association between pain and FMS functional capacity were pain catastrophizing and depression. Both, pain catastrophizing and depression were key factors mediating the association between clinical pain (total and intensity) and FMS functional capacity. The authors discussed the relevance of targeting depression and pain catastrophizing as treatment goals to reduce the impact of pain in FMS patients” daily function. The third study [10] demonstrated the relevant impact of social support on the cognitive performance of FMS patients. All dimensions of the Social Support Behaviours Scale (i.e., emotional support, practical assistance, socializing, financial assistance, advice/guidance, family support, and friends support) exhibited a positive impact on verbal memory, organization and planning abilities, strategic planning, self-regulation, processing speed, attention, and cognitive flexibility of FMS patients. Social support dimensions not only positively impact the number of correct responses and processing speed of neuropsychological tests but also the number of errors.

The fourth study [14], explored descriptively the association between self-efficacy and headache impact, anxiety, and physical activity levels in patients with CTTH; a chronic pain pathology and the most prevalent primary headache disorder worldwide [16, 17]. In this study [14] physical activity levels showed positive moderate correlations with self-efficacy in the domain of physical function. Linear regression models determined that self-efficacy and anxiety sensitivity showed a significant association with the headache impact and the anxiety sensitivity index. In addition, no associations were reported between pain intensity, duration, or frequency and psychosocial factors or headache impact. This study showed a great disease's negative impact on daily tasks and physical activity in CTTH patients. The last factors were influenced by anxiety and self-efficacy.

To conclude, the future of chronic pain prevention, diagnosis, and treatment implies overcoming some relevant concerns and considering some recommendations, such as: (1) the screening for cognitive deficits as a part of the routine diagnostic of FMS, (2) the design of psychoeducation and intervention programs directed not only to FMS patients but also relatives, healthcare workers, and the general population, (3) the use of indices of RT variability, in particular those derived from the ex-Gaussian function, as a complement to assess cognitive function impairments in FMS patients, and (4) the relevance of targeting factors, such as the depression and pain catastrophizing in FMS patients' intervention, and physical activity and self-efficacy in the CTTH patients' intervention; in both conditions with the clinical goal of reducing the disease's impact. The special issue findings highlight the benefits that would entail the creation of personalized evaluation and treatment plans—embracing all the reported factors—in chronic pain patients [18, 19].

*Carmen M. Galvez-Sánchez* *Lorys Castelli* *Casandra I. Montoro*

## References

[1] Gelonch O., Garolera M., Valls J., Rosselló L., Pifarré J. (2017). Cognitive complaints in women with fibromyalgia: are they due to depression or to objective cognitive dysfunction?. *Journal of Clinical and Experimental Neuropsychology*.

[2] Seoane-Mato D., Sánchez-Piedra C., Silva-Fernández L. (2019). Prevalence of rheumatic diseases in adult population in Spain (EPISER 2016 study): aims and methodology. Prevalencia de enfermedades reumáticas en población adulta en España (estudio EPISER 2016). Objetivos y metodología. *Reumatologia clinica*.

[3] Marques A. P., Santo A. S. D. E., Berssaneti A. A., Matsutani L. A., Yuan S. L. K. (2017). Prevalence of fibromyalgia: literature review update. *Revista Brasileira De Reumatologia*.

[4] Breivik H., Collett B., Ventafridda V., Cohen R., Gallacher D. (2006). Survey of chronic pain in Europe: prevalence, impact on daily life, and treatment. *European Journal of Pain*.

[5] Cohen S. P., Vase L., Hooten W. M. (2021). Chronic pain: an update on burden, best practices, and new advances. *Lancet*.

[6] Christopher M. J. (2011). It’s time for bioethics to see chronic pain as an ethical issue. *The American Journal of Bioethics*.

[7] Faustino B. (2021). Transdiagnostic perspective on psychological inflexibility and emotional dysregulation. *Behavioural and Cognitive Psychotherapy*.

[8] Amici P. (2021). Intolerance of uncertainty: from transdiagnostic model to clinical management. *Psychiatria Danubina*.

[9] Duschek S., de Guevara C. M. L., Serrano M. J. F., Montoro C. I., López S. P., Reyes Del Paso G. A. (2022). Variability of reaction time as a marker of executive function impairments in fibromyalgia. *Behavioural Neurology*.

[10] Galvez-Sánchez C. M., Reyes Del Paso G. A., Montoro C. I. (2022). Revealing the role of social support on cognitive deficits in fibromyalgia syndrome. *Behavioural Neurology*.

[11] Montoro C. I., Galvez-Sánchez C. M. (2022). The mediating role of depression and pain catastrophizing in the relationship between functional capacity and pain intensity in patients with fibromyalgia. *Behavioural Neurology*.

[12] Wolfe F., Clauw D. J., Fitzcharles M. A. (2010). The American College of Rheumatology preliminary diagnostic criteria for fibromyalgia and measurement of symptom severity. *Arthritis Care and Research*.

[13] Wolfe F., Smythe H. A., Yunus M. B. (1990). The American College of Rheumatology 1990 criteria for the classification of fibromyalgia. *Arthritis and Rheumatism*.

[14] González de la Flor Á., Pérez G., de Sevilla G. (2022). Relationship between self-efficacy and headache impact, anxiety, and physical activity levels in patients with chronic tension-type headache: an observational study. *Behavioural Neurology*.

[15] Fan Y., Kong X., Liu K., Wu H. (2022). Exercise on striatal dopamine level and anxiety-like behavior in male rats after 2-VO cerebral ischemia. *Behavioural Neurology*.

[16] Stovner L. J., Hagen K., Jensen R. (2007). The global burden of headache: a documentation of headache prevalence and disability worldwide. *Cephalalgia: An International Journal of Headache*.

[17] Headache Classification Committee of the International Headache Society (IHS) (2013). The International Classification of Headache Disorders, 3rd edition (beta version). *Cephalalgia: An International Journal of Headache*.

[18] Braš M., Dorđević V., Milunović V., Brajković L., Miličić D., Konopka L. (2011). Person-centered medicine versus personalized medicine: is it just a sophism? A view from chronic pain management. *Psychiatria Danubina*.

[19] Bruehl S. (2015). Personalized pain medicine: pipe dream or reality?. *Anesthesiology*.

